# Statistical Analysis of the Main Configuration Parameters of the Network Dynamic and Adaptive Radio Protocol (DARP)

**DOI:** 10.3390/s17071502

**Published:** 2017-06-26

**Authors:** Francisco José Estevez, Jesús González, Peter Glösekötter, Olga Valenzuela, Ignacio Rojas

**Affiliations:** 1 Department of Electrical Engineering and Computer Science, University of Applied Sciences of Münster, Stegerweldstr. 39, 48565 Steinfurt, Germany; peter.gloesekoetter@fh-muenster.de; 2 Department of Computer Architecture and Technology, University of Granada, Periodista Daniel Saucedo Aranda S/N, 18071 Granada, Spain; jesusgonzalez@ugr.es (J.G.); irojas@ugr.es (I.R.); 3 Department of Applied Mathematics, University of Granada, Campus Fuentenueva, S/N, 18071 Granada, Spain

**Keywords:** wireless sensor networks (WSN), DARP, routing protocol, ANOVA, fine-tuning configuration

## Abstract

The present work analyses the wireless sensor network protocol (DARP) and the impact of different configuration parameter sets on its performance. Different scenarios have been considered, in order to gain a better understanding of the influence of the configuration on network protocols. The developed statistical analysis is based on the method known as Analysis of Variance (ANOVA), which focuses on the effect of the configuration on the performance of DARP. Three main dependent variables were considered: number of control messages sent during the set-up time, energy consumption and convergence time. A total of 20,413 simulations were carried out to ensure greater robustness in the statistical conclusions. The main goal of this work is to discover the most critical configuration parameters for the protocol, with a view to potential applications in Smart City type scenarios.

## 1. Introduction

Nowadays, a large amount of new technologies are arising in the Smart Cities field [1]. This topic is currently trendy, not only for academia, but also for hundreds of projects all around the world [2,3]. Due to this global movement pushing wireless communications to improve protocols and standards, new ones have emerged.

Within these new network protocols, different have issues appeared, some of which are already solved, but others are not. Responses against adverse events, formation time, energy consumption or message overhead are some examples of issues that still could be improved. IEEE 802.15.4 [4] is the basis for most of the wireless sensor networks (WSN) alternatives applied to Smart Cities. Some, such as the IPv6 Routing Protocol for Low-Power and Lossy Networks (RPL) [5], Dynamic and Adaptive Routing Protocol (DARP) [6] or Ad-Hoc On demand Distance Vector (AODV) [7], present reliable features, but also different disadvantages.

The present work considers Smart Cities as its main objective, thus this work mainly considers cluster-based network protocols [8,9]. Regardless of the network protocol type, network protocols usually focus on the optimization of at least one parameter, e.g., traffic contention, quality of service (QoS), range maximization [10] or energy consumption.

Cluster-based network protocols rely on different key characteristics such as different node types and distance. IEEE 802.15.4 is the most used standard in WSNs and within it, two different node roles are defined: full-function devices (FFD), which are devices with all the routing and storing capabilities; and reduced-function devices (RFD), which are network devices like sensors or end-points that do not need to perform complex tasks.

In cluster-based networks, the main challenge is the selection of the cluster-head. This process becomes critical due to the importance of cluster-heads in the network. Cluster-heads carry out the tasks of message distribution and cluster management.

Among the Internet of Things (IoT), different new protocols have emerged, one of them and the focus of study of this work is the Dynamic and Adaptive Radio Protocol (DARP), which has several advantages and improvements as presented by Estévez et al. in [7,11]. Based on the network layer and especially on network protocols, there is a problem that recurrently emerges, which is the problem of the proper configuration. Most of the modern protocols are able to perform a vast majority of operations in many different scenarios, but this is far from being optimal for a scenario or use case [12,13,14], so we have developed the present work in order to find an optimal configuration for DARP-based networks in Smart City scenarios,

Due to its design, DARP relies on different key parameters, which greatly determine the behavior of the protocol and the performance in a certain scenario. These critical parameters are four timers and four thresholds, being the process of finding their optimal configuration the main goal of this work.

The paper is structured as follows: Section 2 presents the DARP network protocol with its main characteristics. Section 3 presents the simulation framework, the results and the methodology applied to find the main configuration parameters. Section 4 delves into the statistical analysis carried out. Finally, Section 5presents the most relevant conclusions of the present work.

## 2. DARP Overview

**Figure 1:**
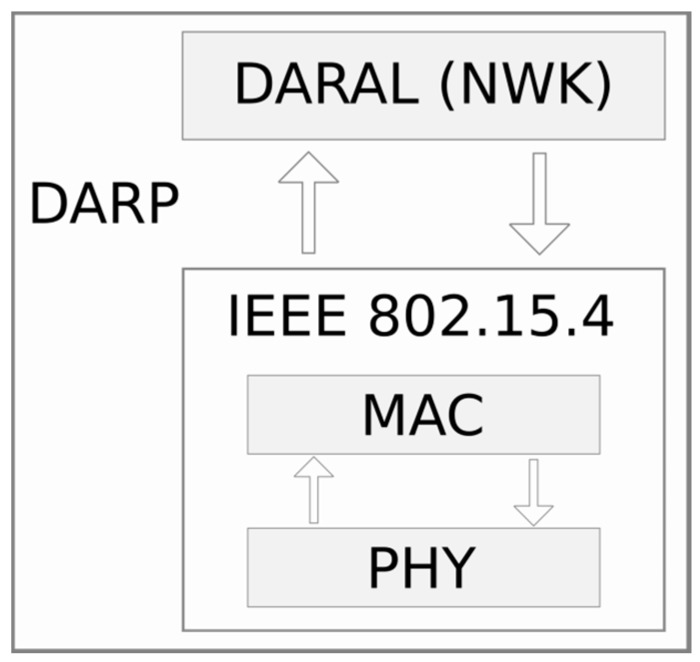
DARP Layer Structure.

The goal of this paper is the development of a fine-tuning configuration method for the DARP protocol [15]. Figure 1 shows this third layer protocol on top of the IEEE 802.15.4 standard (MAC and PHY layers). This section describes the main insights of this protocol.

DARP has been specifically developed for Smart Cities, a large-size scenario type under low-/ medium-density conditions, considering only the infrastructure for public services. DARP is based on a pair of algorithms, the Dynamical and Adaptive Routing Algorithm (DARAL), which carries out the routing metrics and the transport of the packets, and the Dynamical Role Selection Process (DRSP) one, which selects the cluster-head and forms the cluster itself.

**Figure 2:**
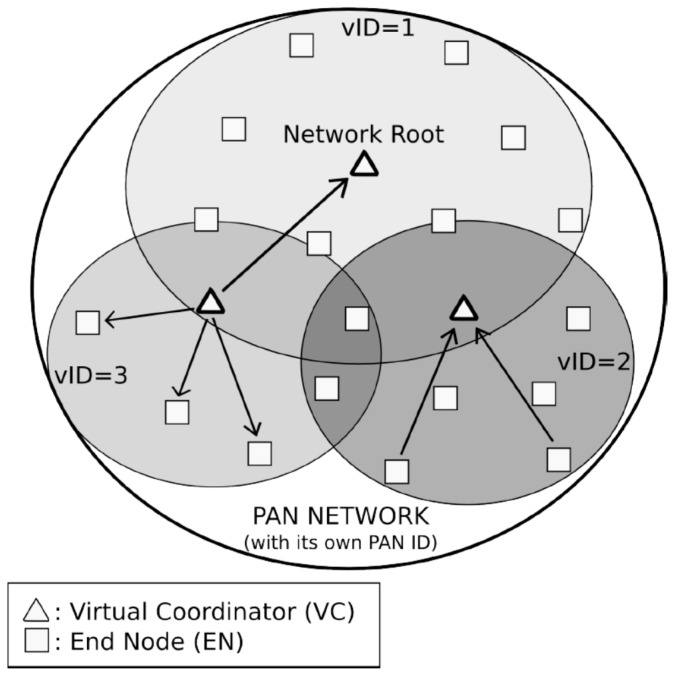
Example of PAN network based on DARAL.

Before entering into a detailed description, it is necessary to define some general concepts about DARP and DARAL, like the sub-network concept and the different types of role. The sub-network concept comes from the clustering techniques and the idea of Smart Cities’ organization. In Smart City approaches, nodes usually communicate in their neighborhood. Thus, if clustering is applied, traffic can be contained in a determined area, allowing the reduction of interference with other nodes and also minimizing the necessity for looking for a node among the complete network topology. In order to improve the clustering concept, the virtual sub-network concept extends this idea, applying it in routing tasks. Each virtual sub-network is identified with a virtual identification (vID), which is used to route messages among the network tree. Figure 2 shows a global Personal Area Network (PAN) with its own identifier, but internally sub-divided into three different sub-networks. The first one is where the network root is located, while the other two group different nodes.

Another general concept about DARAL is the node role, considering that every node should be similar in terms of hardware. DARAL runs a dynamical role selection process (DRSP) at the start-up of every node, which allows it to select the role to be played by a node in the network.

Figure 2 shows the two available roles in DARAL, end node (EN) and virtual coordinator (VC). ENs are nodes that only communicate with the VC of its sub-network. VCs have the same functionality that ENs, but also manage virtual sub-networks and store routing tables.

**Figure 3:**
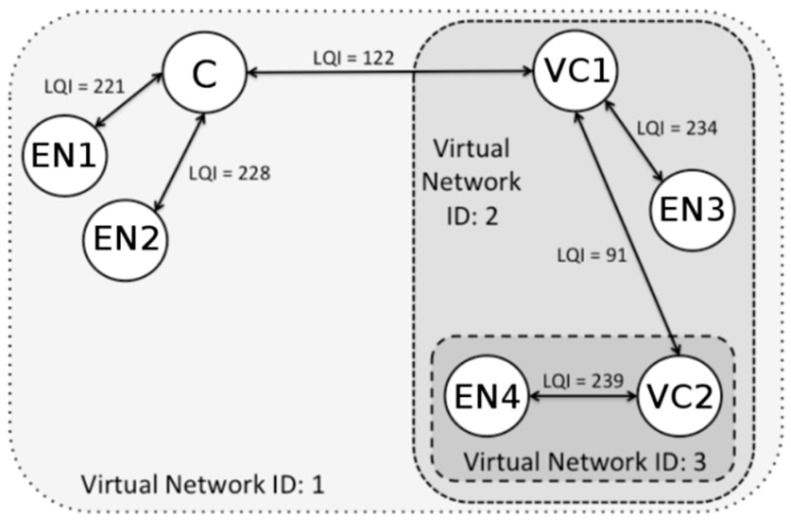
DARP basic network overview.

**Figure 4:**
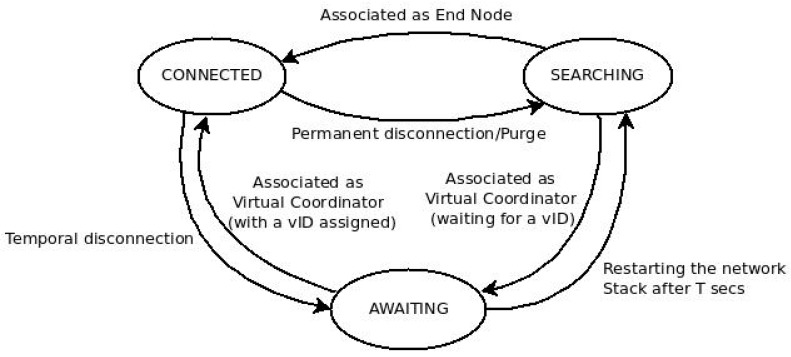
DARP state machine.

Figure 3 shows the application of clustering through the use of parallel and independent virtual sub-networks, with their own network identification (vID). DARP uses the same standard-type of node (IEEE 802.15.4 FFD), creating roles and changing between them in a software-based method. The main role is the virtual coordinator (VC), which carries out the function of cluster-head, managing its own virtual sub-network and delivering inter-/intra-cluster packet sending. The other role is end node (EN), which executes non-cluster-head routines. Thus, it is a node type that only communicates with the VC in the same virtual network, in an intra-cluster communication type. This is a mechanism to reduce the energy used, via reducing the duty cycle. These roles are managed through a state machine, which relies on three different states as shown by Figure 4. DARP shows its better performance during the set-up phase, thus based on its design, it reduces the energy consumption, the convergence time and the number of control messages sent.

The set-up phase is a very important stage that has not been deeply studied due to different factors, but it is indeed a very important phase because it is the moment when a node is characterized and adopts a role. Not only from a node perspective, it is important the set-up phase, but also from a cluster perspective, because the sub-networks are created and the nodes join the different sub-networks.

In the DARP protocol, DRSP performs the action of creating the sub-networks. This algorithm uses three configuration parameters: TH_baselevel_, TH_role_ and L_request_, which greatly characterize the performance of the algorithm. In addition to these parameters, the LQI itself is another key parameter, because it is the representation of the link between a VC and a certain node, and it is vulnerable to interferences in frequency and time domain. In order to avoid these effects, it is possible to apply different methods, e.g., sampling the LQI during a certain time frame, minimizing short-scale effects. Other possibility that does not impact the convergence time is the use of different MAC layers, like the improved MAC proposed by Kobatake et al. [16], where they proposed a MCR-SS-CSMA/CA alternative for reducing interferences. Another possibility in this context is the contribution of Koster et al. [17], where they developed a method for reducing the interferences in hostile industrial environments, which could be also applied in other environments.

Once a node receives an association response message from the cluster-head (VC), it sets the T_link_ timer. After expiring, DRSP is executed, by analyzing the different responses from all the VCs in range that have answered with an association response. If the LQI of the answer was beyond TH_baselevelv_, the VC is discarded as candidate. Otherwise, the VC becomes a candidate that can be picked up by *DRSP*, which is going to look for the response with the best LQI. Upon the best response is selected, then it is compared with TH_role_ and it checks if the number of collected requests exceeds L_request_, thus the node configures itself as VC. If the collected requests do not exceed L_request_, the node compares the best LQI with TH_role_ and if it is higher than this threshold, it configures itself as EN; otherwise it configures itself as VC.

If a certain node cannot find a valid cluster-head to connect with it, it sets T_reconnect_ and waits until it expires, to resend another association request. In addition to DRSP, DARAL includes additional parameters to control the density and number of nodes per cluster, e.g., L_nodes_, which is used to decide whether a VC answers with an association request allowing a new node connect the virtual-network or not. Once the node is connected, DARAL sets on different timers, T_down_ is used when a node does not answer to a message, thus, this finishes in its drop from the virtual-network. T_ack_ controls the time frame within a node can answer to a message acknowledging the reception.

The configuration parameters and their functions are depicted below:

- TH_baselevel_: The base level threshold represents the minimum LQI that a link between two nodes can have. If the link is not above this threshold, the connection is discarded.
- TH_role_: The role in the network is based on the role threshold. If LQI is above TH_role_, a node connects as EN, otherwise as VC.
- L_nodes_: The maximum number of nodes in a virtual network is set by the node limit parameter, balancing the load between different virtual networks.
- L_request_: The request limit is a parameter that sets the number of association requests that a certain node can hear before setting its own role to cluster-head. Before the node is connected and DRSP launched, the node can hear different messages from its neighbors. If the association request exceeds this parameter, when *DRSP* is fired, the node configures itself as cluster-head.
- T_link_: The awaiting time for receiving more responses from different VCs is defined by the timer for link evaluation. After expiring the timer, the connection process begins.
- T_down_: A certain node exceeding this timer without answering messages, it is going to be removed from the virtual network, spreading the information among the VCs in the network.
- T_reconnect_: The reconnection timer has two different purposes. First, it is used by a VC without vID to resend the vID request to its VC. Second, it is used by non-connected nodes to restart the network stack, restarting also the whole connection process.
- T_ack_: If an ACK is not received before the timer for acknowledgement expires, then the original message should be resent again.

## 3. DARP Performance

The following section depicts the methodology used in the simulations, including also the environment and the conditions applied to find an optimal configuration of DARP for Smart City scenarios.

### 3.1. Simulations and Evaluation Scenarios

The solution used for carrying out the simulations has been OMNeT++ [18]. This simulator is an event-based simulator and it has been widely used by researchers around the world. OMNeT++ is not a specific wireless simulator, therefore it is necessary to combine it with a proper framework, which in the present work has been Inetmanet [19]. The combination of OMNeT++ and Inetmanet has been used for the development and test of many different protocols, DARP [20] among them.

The main objective of this study are Smart City type scenarios [2,3,11], thus the scenario for testing has been configured as a Smart City in terms of density, using static randomly distributed nodes. Other details such as communication band or network type are defined following the requirements of using the IEEE 802.15.4 standard, which limits them to a few possibilities the variability of configurations. Because of this fact, the 2.4 GHz ISM band and a beaconless network have been used for the present work. The other modules in the simulation framework: battery, physical (PHY) layer, medium access (MAC) layer and application (APL) layer, also need some basic configuration, which is showed in Table 1.

Previous works such as [11,21] have defined a medium node density (ND = 10) as the most interesting one for Smart City type scenarios, where the infrastructure is not a dense. Based on the analytical purpose of this work, medium-density scenarios are a considered to be dense enough.

However, the performance of the DARP methodology can be influenced by the different values of the parameters defining it (mainly: the Role Threshold (TH_R), the ACK Timer, (T_ACK), the Baselevel Threshold (TH_B), the Reconnection Timer (T_REC), the Down Timer (T_DOWN) and the Link Timer (T_LINK). Therefore, it is necessary a statistical analysis in order clearly understand the behavior of *DARP*.

### 3.2. Statistical Analysis of DARP

The Analysis of Variance (ANOVA) tool is an extended and widely used statistical method [22,23,24,25]. The ANOVA analysis is a powerful statistical tool that allows to contrast the null hypothesis that the means of several populations are equal (in our analysis, the value of the dependent variable when the following relevant factors are modified: the Baselevel Threshold (TH_B), the Role Threshold (TH_R), the ACK Timer (T_ACK), the Reconnection Timer (T_REC), the Down Timer (T_DOWN) and the Link Timer (T_LINK)) against the alternative hypothesis that at least one of the populations differs from the others in terms of their expected value. This contrast is fundamental in the analysis of experimental results, in which it is interesting to compare the results of several factors with respect to the dependent variable.

For the scenarios considered in this work, which have a certain fixed complexity, such the one commented in Section 3.1. It is necessary to carry out some adaptation in the parameters according to the different levels shown in Table 2.

The necessity of carrying out a precise analysis, lead us to use ANOVA, where a large set of samples is considered, in order to verify and analyze the performance of DARP under different conditions. This large set of samples requires a high number of simulations, which by extension requires a very high computational time (exponentially scaling up to an unmanageable level). In order to control and reduce this large number, a fixed set of levels for every factor have been considered, selecting significant enough values from previous analysis [11]. The results obtained from the statistical analysis provide a good insight in the performance of DARP in Smart Cities. The following section delves into the results of the statistical analysis, presenting the main results.

### 3.3. Statistical Analysis as a Tool for Analyzing Other Network Protocols

The statistical tool (ANOVA) used in this paper can be also applied to evaluate the behavior of other relevant protocols presented in the bibliography. In this sense it is important, for example, to mention the AODV protocol [26]. The AODV protocol has several parameters (such as Route Cost, RREP generation timer, minimum number of hops, valid route time, queue length, traffic direction, router type, traffic density, delay, sequence number information, etc.) which can be analyzed with the methodology presented in this contribution in order to better understand the behavior of this protocol (e.g., in terms of packet delivery rate, routing overhead, energy consumption, etc.).

Another protocol of relevance presented in the bibliography is RPL-6LoWPAN [27]. This protocol has also relevant functions (objective function), strategies/mechanisms (such as collision avoidance mechanism) and parameters (such as DODAG timer, join time, ACK timer, etc.) whose study, performing a similar statistical NOVA analysis, may be relevant to determine the protocol’s performance and its adaptability to different scenarios.

Recently, and with the great interest of Internet-of-Things (IoT) and using IPv6 as communication technology, has appeared the Thread protocol [28], where the parameters that may have more relevance on the behavior of the system (and therefore susceptible to a statistical study) can be minimum and maximum data message interval time for the trickle algorithm, trickle expiration timers or ACK timer.

## 4. Results of the Statistical Analysis of DARP

The first step to perform the statistical study is to determine the different levels that the main factors can have. We must keep in mind in our experiments, that we have quantitative variables, and not qualitative ones. Therefore, certain intervals for each of the analyzed factor should be defined (what we call levels). In order to have a first approximation of the statistical relevance of the selected factors, a division into three levels or intervals have be made for each of these factors. Table 2 presents the three intervals (without intersection between them) in which the quantitative variables were characterized (the main factors) in order to carry out the ANOVA statistical study.

The second step is to determine the output variable (which characterizes the behavior of the DARP system [15]). In this sense and analyzing the bibliography [11,21], the dependent variables selected. To study the behavior of the DARP system has been: (a) Number of control messages sent during the set-up time; (b) Set-Up Energy and (c) Convergence Time (s). It is relevant to remark that the main task is to determine how the output variables (in our case these dependent variables are focused in energy consumption of the net and time required for convergence) are influenced when there are variations in the choice of the different levels of the main factors.

In order to have greater robustness in the statistical conclusions of the present study, all experiments were repeated 10 times. That is, for each of the possible configurations of the different levels of the main factors, a total of 10 executions were made, measuring the dependent variables.

### 4.1. Statistical Analysis of the Number of Control Message

An ANOVA study was performed simultaneously analyzing the six main factors and allowing the interaction between them. As indicated in the previous section, for each of the factor configurations, multiple simulations were performed (a total of 20,413 simulations were carried out). Table 3 shows the results for the dependent variable Number of control messages sent during the set-up time.

Table 3 shows, for each of the factors and for the significant interactions, the sum of squares (a measure that represents the variation or deviation with respect to the mean, and calculated as a sum of the squares of the differences with respect to the mean), degrees of freedom (difference between number of observations and parameters to be estimated), mean square (that represents an estimate of the variance of the population) and the F-Ratio (significance of the factor under study) and *p*-value(which corresponds to the lowest possible level of significance that can be chosen, for which the alternative hypothesis would still be accepted with the current observations). From the data and information presented in the Table 3, the statistical test F-Ratio and the associated *p*-value are the most relevant ones.

Generally, when the *p*-value associated with the F-Ratio value is smaller than 0.05, then the null hypothesis must be rejected and should be accepted that there are differences between the means of the different factor levels (or in our case of interactions) selected. Analyzing the *p*-value of Table 3, it can be concluded that there are three factors, whose choice among the three different values of their levels, significantly affect the variable Number of Control Message. These factors, ordered by their relevance, are: Reconnection Timer (T_REC), ACK Timer and Link Timer. Regarding the relevant interaction, it is important to comment the interaction between the Reconnection Timer (T_REC) and ACK Timer, and the interaction between Reconnection Timer and Link Timer (for reasons of space and compression, only the most significant interactions are shown).

The main objective studying the behavior of the different factors by ANOVA, was to know if these levels differ significantly from each other and therefore have an impact on the dependent variable. Once detected the most relevant factors and the existence of differences between the effects of the factor, it is important to know which specific levels produces the greatest effect or which are different/equal levels of each factor. To solve this question, additional comparisons can be made between groups of means factors, by using Multiple Ranges Test. The method used to discriminate between means is Fisher’s least significant difference (LSD) procedure, which is based on the construction of hypothesis tests for the difference of any pair of means.

**Figure 5:**
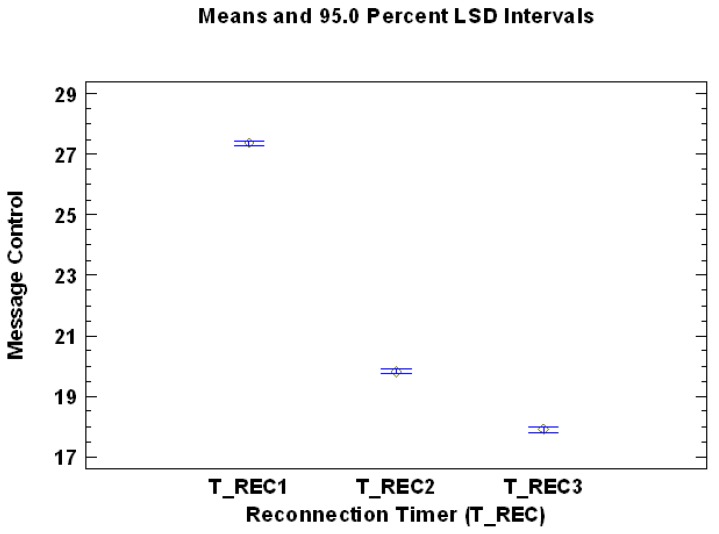
Mean values of the levels of the factor Reconnection Timer, analyzing the dependent variable Number of control messages sent during the set-up time.

Table 4 presents the Multiple Range Test for Reconnection Timer, which is one of the most relevant factors having influence on the variable Number of control messages sent during the set-up time. From Table 4, it can be established that there are three homogenous groups without intersection (identified using columns of X’s), which means that the three different levels of the Reconnection Timer have all significant and different influences on the dependent variable. The level T_REC3 (with values in the interval [11–50]) has the best performance and therefore lowest mean value, and the level T_REC1 (with values in the interval [0.1–4]) has the worst performance with the highest mean value. This information can also be presented graphically (Figure 5).

**Figure 6:**
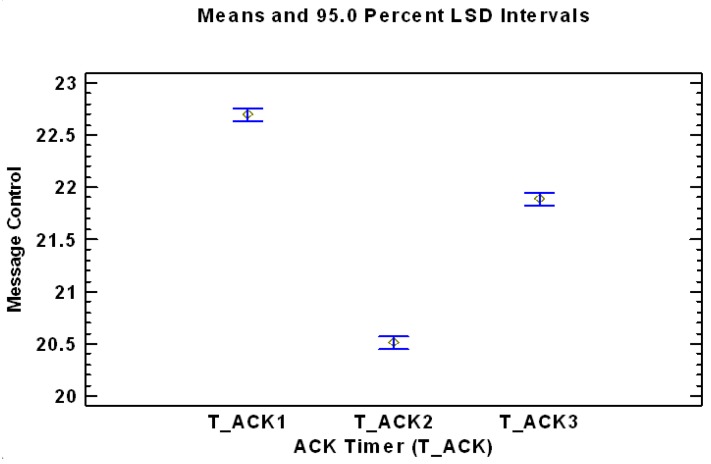
Mean values of the levels of the factor ACK Timer factor, analyzing the dependent variable Number of control messages sent during the set-up time.

A similar analysis can be performed with the ACK Timer factor. In this case, three completely different and statistically significant levels for ACK Timer factor are presented in Table 5 and graphically in Figure 6.

**Figure 7:**
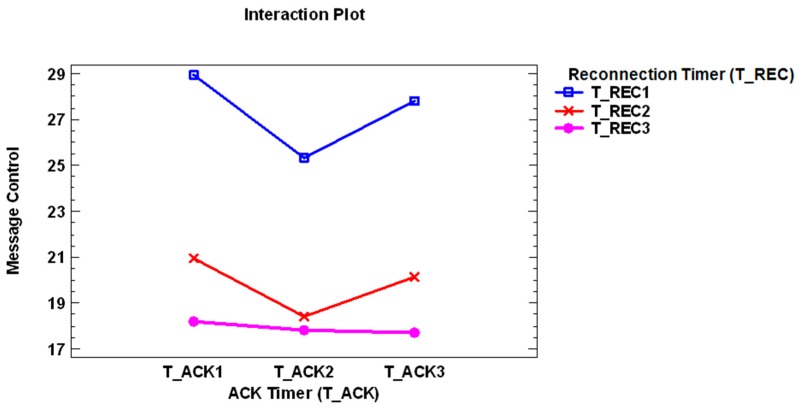
Evolution of the interaction between the main factor Reconnection Timer and ACK Timer, analyzing the dependent variable Number of control messages sent during the set-up time.

Regarding the interactions factor, one of the six more representatives is the interaction between the main factor Reconnection Timer and ACK Timer. Figure 7 presents this evolution for the different levels of these factors, showing that a higher time configuration presents a lower use of control message.

### 4.2. Statistical Analysis of the Energy

The second dependent variable that is relevant to be analyzed in the wireless sensor network protocol, DARP, is focused on the energy field. A statistical study, as presented in Section 4.1, was performed for the Set-Up Energy (Energy) variable. As presented in the bibliography [5,6], actual network protocols required energy-efficient wireless routing protocol, and frequently reconstruct the whole net system, meaning that the set-up energy is consistently consumed. Table 6 presents the statistical summary of the ANOVA being the dependent variable Set-Up Energy. There are three main factors: Reconnection Timer, ACK Timer and Link Timer with a *p*-value smaller than 0.05, and therefore the election among the levels of these factors have a strong relevance in the energy behavior of the DARP protocol.

**Figure 8:**
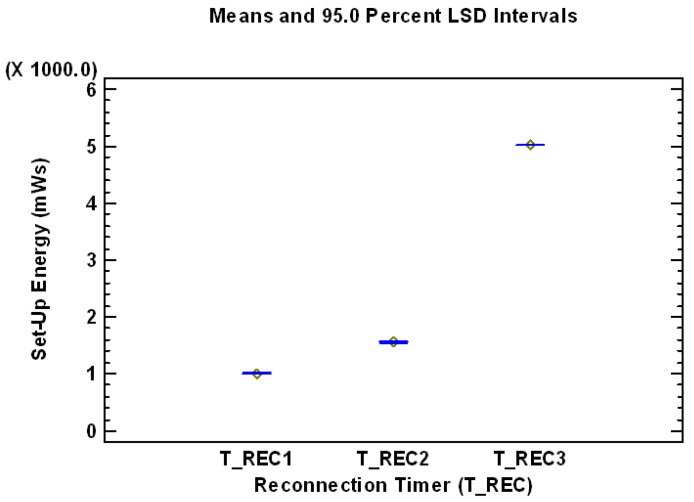
Mean values of the levels of the factor Reconnection Timer, analyzing the dependent variable Set-Up Energy (mWs).

**Figure 9:**
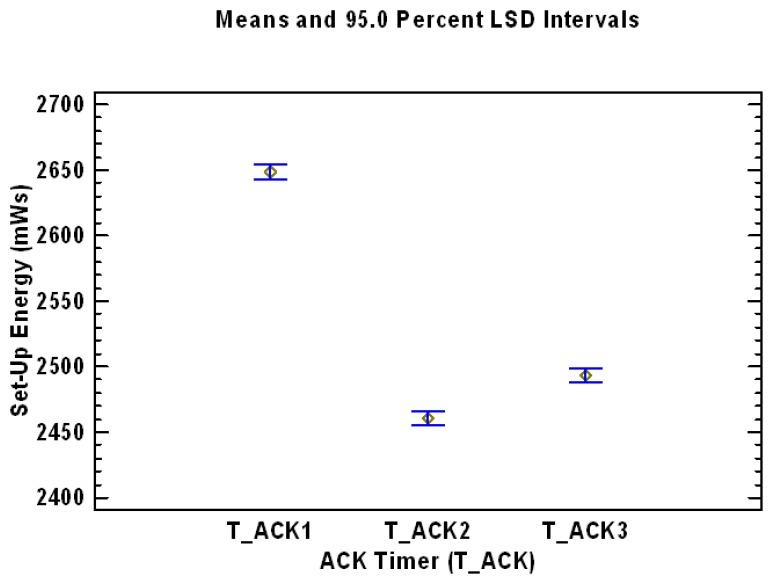
Mean values of the levels of the factor ACK Timer factor, analyzing the dependent variable Set-Up Energy (mWs).

Table 7 and Figure 8 present the Multiple Range Test for Reconnection Timer using as dependent variable the Set-Up Energy (mWs). As can be concluded from Table 7, there are three homogenous groups without intersection (identified using columns of X’s), which means that the three different levels of the factor Reconnection Timer have all of them statistically significant effect on the dependent variable Set-Up Energy. The level T_REC1has the best performance and therefore lowest mean value in term of required Energy, and the level T_REC3 has the worst performance with the highest mean value. It is also important to analyze the levels of the statistically significant factor ACK Timer (T_ACK). The information of the Multiple Range Test for ACK Timer using as dependent variable the Set-Up Energy (mWs) is presented in Table 8 and graphically in Figure 9, which shows the evolution of the mean values of the levels of the factor Reconnection Timer being the dependent variable Set-Up Energy (mWs).

### 4.3. Statistical Analysis of the Convergence Time

In the analysis of a routing algorithm for wireless sensor networks, the development of a best-link selection algorithm based on the quality of the links, which allows dynamically adjusting the role of a node in the routing algorithm, it is important to minimize the message overhead, energy consumption and also convergence time, during the formation phase. Therefore, in this subsection the evolution of the Convergence Time is analyzed when the levels of the factor presented in Table 2 are modified, using the same methodology presented in Section 4.1 and Section 4.2

The same conclusion, as the obtained from previous statistical analysis, can be summarized from Table 9, the three factors: Reconnection Timer, ACK Timer and Link Timer, have levels with strong influence in the behavior of the Convergence Time of the DARP system. However, it is also relevant to take into account, that there are other factors with are statistical significant: Baselevel Threshold and Role Threshold. Table 10 presents the result of the multiple comparison procedure to conclude which levels are significantly different from which others for Reconnection Timer factor, when the output variable is the Convergence Time. As can be observed, the three factors are statistically different. A similar conclusion can be obtained for factor ACK Timer in Table 11.

## 5. Conclusions

The present work has analyzed the performance of the DARP network protocol for large-size Smart City type scenarios under low and medium density conditions, focusing on the analysis of the configuration parameters.

The protocol DARP is based on two algorithms, DRSP and DARAL, which carry out the task of communication within large communication infrastructures. The core of this proposal relies on a link quality indicator based method for selecting the best link, allowing a node to choose its own role in the network, as well as the cluster to connect with. These characteristics have been demonstrated effective to reduce the control message overhead, the energy consumption and the convergence time during the network set-up phase.

This network protocol has been already presented in prior work but its performance needs to be analyzed with different relevant factors in the scope. The factors that have been considered are: the Reconnection Timer (T_REC), the ACK Timer (T_ACK), the Link Timer (T_LINK), the Baselevel Threshold (TH_B), the Down Timer (T_DOWN) and the Role Threshold (TH_R). In order to characterize the performance of the network, some criterion has been defined, based on previous work. This criterion is characterized by the number of control messages sent during the set-up time, the set-up energy and the convergence time. A good understanding of the performances of the alternative operators leads to the improvement of the DARP protocol itself. The importance of the operators involved in DARP is analyzed using ANOVA, due to its valuable results, as it has been shown in the bibliography.

Among all the configuration set of parameters, ACK Timer (T_ack_), Link Timer (T_link_) and Reconnection Timer (T_reconnect_) have emerged as the most important configuration parameters for the set-up phase. The results show that these timers impact the overall energy consumption, convergence time and number of control messages sent. On the other hand, for long-time simulations, ACK timer (T_ack_), Role Threshold (TH_role_) or Baselevel Threshold (TH_baselevel_) seem to be more sensitive to the overall performance, which might require future studies as well.

## Figures and Tables

**Table 1:** Main configuration parameters for the battery module, PHY, MAC and APL layers.

Configuration	Value
Parameter	
Carrier Frequency (GHz)	2.4
Carrier Sense Sensitivity (dBm)	−85
Transmit Power (mW)	1.0
Sensitivity (dBm)	−85
Thermal Noise (dBm)	−110
SNR Threshold (dB)	4
Path Loss Alpha	3
T_alive_ (s)	600
Payload Size (Bytes)	70
Maximum Simulation Time (s)	1200
CPU Active Drain (mA)	7.6
CPU Sleep Drain (mA)	0.237
CPU Radio Idle State (mA)	1.38
CPU Radio Detection (mA)	9.6
CPU Radio Sleep State (mA)	0.06
Battery Capacity (mA)	1500
Battery Voltage (v)	5.0

**Table 2:** Selection of the different intervals or levels in which the different numerical values of the main factors were categorized to analyze the behavior of the DARP system.

Levels of the Factors			
Factors	Level 1	Level 2	Level 3
Baselevel Threshold(TH_B)	TH_B1/1–10 (5)	TH_B2/11–50 (30)	TH_B3/51–150 (100)
Role Threshold (TH_R)	TH_R1/75–125 (100)	TH_R2/126–175 (150)	TH_R3/176–225 (200)
ACK Timer (T_ACK)	T_ACK1/0.1–1.0 (0.5)	T_ACK2/1.1–2.0 (1.5)	T_ACK3/2.1–10 (6.0)
Reconnection Timer (T_REC)	T_REC1/0.1–4 (2)	T_REC2/4.1–10 (6)	T_REC3/11–50 (30)
Down Timer (T_DOWN),	T_DOWN1/ 0.1–10 (5)	T_DOWN2/11–100 (50)	T_DOWN3/101–200 (150)
Link Timer (T_LINK)	T_LINK1/0.1–2 (1)	T_LINK2/2.1–10 (6)	T_LINK3/11–20 (15)

**Table 3:** Summary of the ANOVA table, which decomposes the variability of Number of control messages sent during the set-up time into contributions due to several factors. The main factors marked with * are statistically significant. Because the interactions between factors have been allowed in the ANOVA, the statistically relevant interactions are also marked with *.

Source	Sum of Squares	Df	Mean Square	F-Ratio	*p*-Value
MAIN EFFECTS					
A: Baselevel Threshold (TH_B)	81.7	2	40.85	2.91	0.0545
B: Role Threshold (TH_R)	31.6	2	15.80	1.13	0.3244
C: ACK Timer (T_ACK) *	1.63 × 10^4^	2	8.1 × 10^3^	580	0.0000
D: Reconnection Timer (T_REC) *	3.36 × 10^5^	2	1.68 × 10^5^	1.20 × 10^4^	0.0000
E: Down Timer (T_DOWN)	3.99	2	1.99	0.14	0.8675
F: Link Timer (T_LINK) *	4834	2	2.41 × 10^3^	172	0.0000
INTERACTIONS					
CD *	7093	4	1.77 × 10^3^	126	0.0000
CF *	4660	4	1.16 × 10^3^	82	0.0000
DF *	6751	4	1.69 × 10^3^	120	0.0000
RESIDUAL	2.85 × 10^5^	20,314	9.77 × 10^4^		
TOTAL (CORRECTED)	7.31 × 10^5^	20,412			

**Table 4:** Results of the Multiple Range Tests for the factor Reconnection Timer (T_REC), using as dependent variable the Number of control messages sent during the set-up time.

Reconnection Timer (T_REC)	LS Mean	LS Sigma	Homogeneous Groups		
T_REC3	17.9	0.045	X		
T_REC2	19.8	0.046		X	
T_REC1	27.3	0.045			X
Limit to establish significant differences: 0.13					

**Table 5:** Results of the Multiple Range Tests for the factor ACK Timer (T_ACK), using as the dependent variable the Number of control messages sent during the set-up time.

ACK Timer (T_ACK)	LS Mean	LS Sigma	Homogeneous Groups		
T_ACK2	20.5	0.046	X		
T_ACK3	21.9	0.046		X	
T_ACK1	22.7	0.045			X
Limit to establish significant differences: 0.13					

**Table 6:** Summary of the ANOVA table, which decomposes the variability Set-Up Energy (mWs) into contributions due to several factors. The factors and interactions marked with * are statistically significant.

Source	Sum of Squares	Df	Mean Square	F-Ratio	*p*-Value
MAIN EFFECTS					
A: Baselevel Threshold (TH_B)	2.30 × 10^5^	2	1.15×10^5^	1.18	0.3080
B: Role Threshold (TH_R)	3.46 × 10^5^	2	1.73 ×10^5^	1.77	0.1702
C: ACK Timer (T_ACK) *	1.35 × 10^8^	2	6.79 ×10^7^	694	0.0000
D: Reconnection Timer (T_REC) *	6.39 × 10^10^	2	3.19 ×10^10^	3.27 × 10^5^	0.0000
E: Down Timer (T_DOWN)	1.62 × 10^5^	2	8.12 × 10^4^	0.83	0.4358
F: Link Timer (T_LINK) *	1.05 × 10^8^	2	5.27 × 10^7^	539	0.0000
INTERACTIONS					
AD *	8.24 × 10^6^	4	2.06 × 10^6^	21	0.0000
BC *	2.60 × 10^6^	4	6.50 × 10^5^	6.66	0.0000
CD *	7.25 × 10^7^	4	1.81 × 10^7^	185	0.0000
CF *	9.88 × 10^5^	4	2.47 × 10^5^	2.53	0.0386
CG *	3.32 × 10^6^	4	8.31 × 10^5^	8.51	0.0000
DE *	1.16 × 10^6^	4	2.92 × 10^4^	2.99	0.0177
DF *	1.89 × 10^7^	4	4.74 × 10^6^	48	0.0000
DG *	2.90 × 10^8^	4	7.27 × 10^7^	744	0.0000
RESIDUAL	1.98 × 10^9^	20,314	9.77 × 10^4^		
TOTAL (CORRECTED)	6.78 × 10^10^	20,412			

**Table 7:** Results of the Multiple Range Tests for the factor Reconnection Timer (T_REC), using as dependent variable the Set-Up Energy (mWs).

Reconnection Timer (T_REC)	LS Mean	LS Sigma	Homogeneous Groups		
T_REC1	1013	3.81	X		
T_REC2	1557	3.87		X	
T_REC3	5031	3.82			X
Limit to establish significant differences: 10.6					

**Table 8:** Results of the Multiple Range Tests for the factor ACK Timer (T_ACK), using as dependent variable the Set-Up Energy (mWs).

ACK Timer (T_ACK)	LS Mean	LS Sigma	Homogeneous Groups		
T_ACK2	2460	3.86	X		
T_ACK3	2493	3.86		X	
T_ACK1	2648	3.78			X
Limit to establish significant differences: 10.6					

**Table 9:** Summary of the ANOVA table, which decomposes the variability Convergence Time (s) into contributions due to several factors. The factors and interactions marked with * are statistically significant.

Source	Sum of Squares	Df	Mean Square	F-Ratio	*p*-Value
MAIN EFFECTS					
A:Baselevel Threshold (TH_B)	2.5 × 10^7^	2	1.26 × 10^6^	9.65	0.0001
B:Role Threshold (TH_R)	1.83 × 10^6^	2	9.17 × 10^5^	7.00	0.0009
C:ACK Timer (T_ACK) *	1.46 × 10^8^	2	7.31 × 10^7^	557	0.0000
D:Reconnection Timer (T_REC) *	6.38 × 10^10^	2	3.19 × 10^10^	2.43 × 10^5^	0.0000
E:Down Timer (T_DOWN)	1.15 × 10^5^	2	5.77 × 10^4^	0.44	0.6435
F:Link Timer (T_LINK) *	1.05 × 10^8^	2	5.27 × 10^7^	403	0.0000
INTERACTIONS					
AD *	1.28 × 10^7^	4	3.22 × 10^6^	24.6	0.0000
CD *	7.77 × 10^5^	4	1.94 × 10^7^	148	0.0000
CF *	3.32 × 10^6^	4	8.32 × 10^5^	6.35	0.0000
DF *	1.92 × 10^7^	4	4.80 × 10^6^	36.63	0.0000
RESIDUAL	2.66 × 10^9^	20,314	9.77 × 10^4^		
TOTAL (CORRECTED)	6.78 × 10^10^	20,412			

**Table 10:** Results of the Multiple Range Tests for the factor Reconnection Timer (T_REC) using as dependent variable Convergence Time.

Reconnection Timer (T_REC)	LS Mean	LS Sigma	Homogeneous Groups		
T_REC1	1011	4.40	X		
T_REC2	1552	4.46		X	
T_REC3	5021	4.41			X
Limit to establish significant differences: 12.2					

**Table 11:** Results of the Multiple Range Tests for the factor ACK Timer (T_ACK) using as dependent variable Convergence Time.

ACK Timer (T_ACK)	LS Mean	LS Sigma	Homogeneous Groups		
T_ACK2	2453	4.45	X		
T_ACK3	2485	4.44		X	
T_ACK1	2647	4.38			X
Limit to establish significant differences: 12.2					
